# A Case Report of a Fulminant Necrotizing Fasciitis of the Upper Extremity in an Immunocompromised Patient

**DOI:** 10.7759/cureus.116591

**Published:** 2026-09-20

**Authors:** Reagan Boyett, Gregory S Morris, Alexandra G Smith, John B Boyett, Joseph P Boyett

**Affiliations:** 1 Medicine, Edward Via College of Osteopathic Medicine, Auburn, USA; 2 Research, Alabama College of Osteopathic Medicine, Dothan, USA; 3 Orthopedic Surgery, Athens-Limestone Hospital, Athens, USA

**Keywords:** immunocompromised status, multidisciplinary management, necrotizing fasciitis (nf), stem cell transplant recipient, upper extremity amputation

## Abstract

Necrotizing fasciitis (NF) is a rare, extremely aggressive, and limb-and-life-threatening soft tissue infection requiring emergent surgical intervention. Diagnosis is often delayed by nonspecific early findings, and immunocompromised patients are at particular risk for fulminant, atypical presentations. We present a case of a 70-year-old male with a remote history of multiple myeloma treated with stem cell transplant who developed extensive NF of the right upper extremity within 24 hours of minor blunt trauma. Despite emergent surgical debridement, the patient progressed to septic shock and ultimately required above-elbow amputation. He was recovering well at the four-month follow-up visit.

This case highlights the diagnostic challenge posed by NF following trivial trauma in patients with occult or remote immunosuppression and underscores the importance of a low threshold for surgical exploration when clinical suspicion is high despite an unimpressive mechanism of injury.

## Introduction

Necrotizing fasciitis (NF) is defined as a rapidly progressive inflammatory infection of the fascia, with secondary invasion of surrounding tissue [1]. NF has rightfully earned the ominous common name of “flesh-eating bacteria”. The most common cause of NF is Streptococcus pyogenes, or group A streptococcus (GAS), but it is often polymicrobial in origin [2]. Patients who develop NF need prompt and aggressive treatment due to the high mortality rate and difficulty to treat if caught late.

The infection causes massive cytokine release, which leads to microthrombosis of surrounding blood vessels. This causes necrosis and a favorable environment for bacterial proliferation because the host immune system has difficulty invading the oxygen-poor environment of necrosed skin [3]. The necrosis creates a breeding ground for endo- and exotoxin-producing bacteria that eventually cannot be suppressed. The most common comorbidities for NF are liver cirrhosis, chronic heart failure, obesity, alcohol abuse, immunodeficiency, systemic lupus erythematosus, Addison’s disease, pre-existing hypertension, and peripheral vascular disease [1]. According to a study done by Misiakos et al., the single most common comorbidity is diabetes mellitus, with 40-60% of patients who develop NF also having diabetes mellitus. The trouble with early detection is that externally, patients may just appear to have cellulitis, which makes it even more vital to be proactive in patients with increased risk [1].

In 2018, the incidence of NF in the United States was estimated to be 33,600 and 28,500 cases according to the National Inpatient Sample and Watson Health datasets, respectively [4]. Amputation rates of NF have been reported to range from 16.8% to 32% [5,6]. Risk factors for amputation include lower extremity involvement, cutaneous and muscle necrosis, diabetes, elevated LRINEC scores, low RBC counts, and decreased albumin [5]. A recent review reported that the majority of patients with NF can trace the origin of the infection to an insult to the extremities instead of the abdomen, of which only 6-27%, depending on the study, involve the upper extremities [7].

In the case of the 70-year-old male presented, he appeared to be immunosuppressed based on his recent history of fungal parasinus infection, though he was not on any chronic immunosuppressive agents during this time. The speed at which this patient’s condition deteriorated despite only having an outpatient surgery over a month ago makes his NF an atypical presentation. This presentation highlights the potential long-term sequelae of a stem cell transplant recipient in an individual with an autoimmune disease.

## Case presentation

A 70-year-old white American male presented to the emergency room with extensive necrosis of the right upper extremity. He has a history of ankylosing spondylitis, Hashimoto autoimmune thyroiditis, juvenile rheumatoid arthritis, and multiple myeloma, for which he underwent a stem cell transplant 16 years prior. He had also had a previous amputation of one of his big toes after trauma from logging during his childhood.

**Figure 1:**
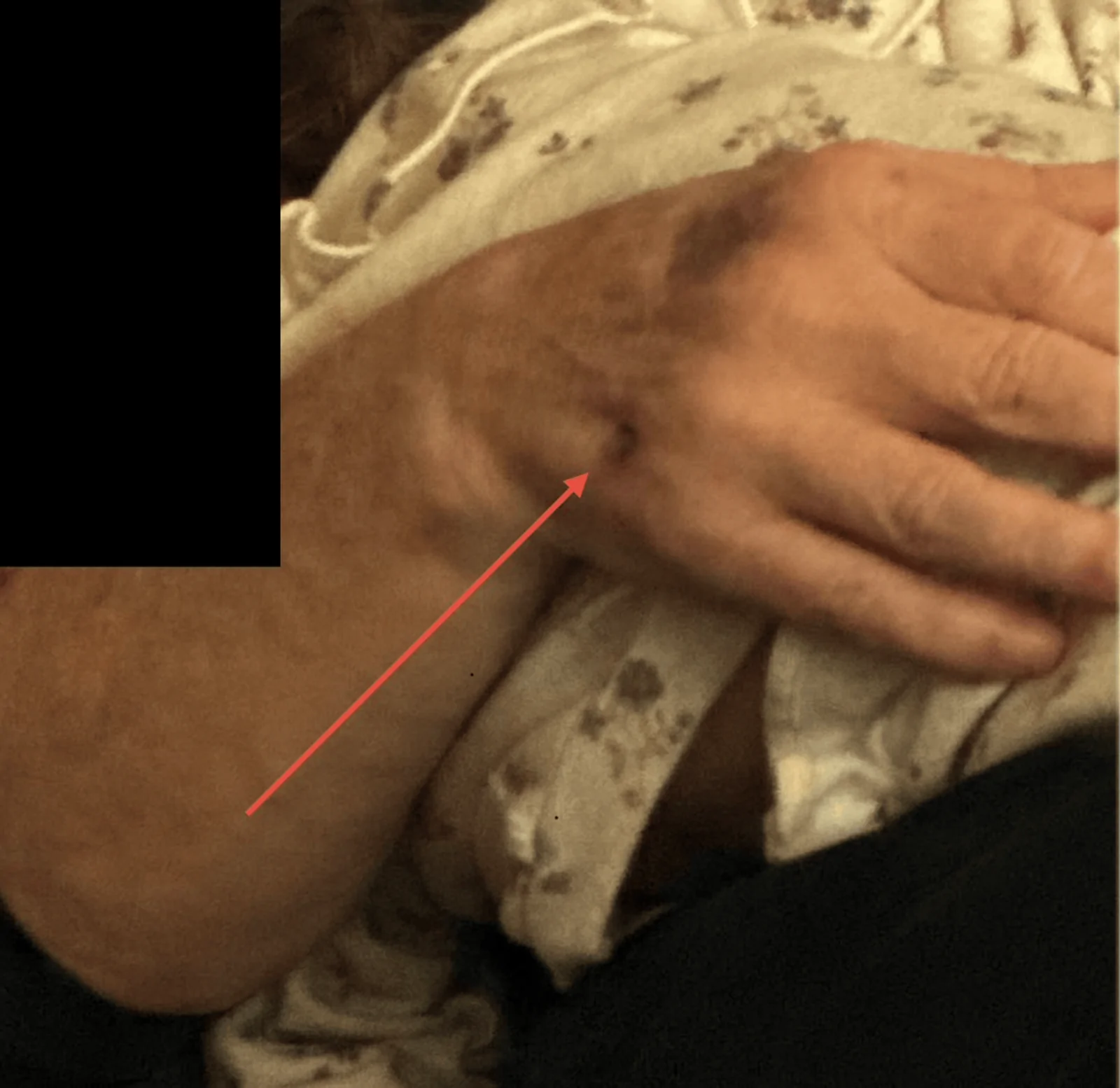
Image of the upper extremity at 5 pm on the day of the injury This image was taken incidentally at around 5 pm on the day of the injury. The red arrow points to the dorsal hand and seeding injury.

The patient reports that the previous morning, he had visited his ENT surgeon for a 6-week follow-up from parasinus surgery, where he received a steroid shot. While in the doctor's office, he bumped his hand on a blunt object and had a small break on his skin over his fifth metacarpal. Throughout the day and evening, the injury remained benign. At 5 pm that day, a photograph was incidentally captured of the injury (Figure 1). At roughly 12 am, the patient began to feel ill and had vomiting, fever, and diarrhea. At 9 am the following day, he was taken to the emergency department via ambulance.

At admission, his vitals showed a low-grade fever of 99.2°F (37.3°C) on temporal scan, tachycardia of 137 bpm, and tachypnea of 29 bpm (Table 1). His labs revealed a low albumin of 2.8 gm/dL, CRP of 6.17 mg/dL, and lactic acid of 6.4 mm/L. Full labs and complete cell count are seen in Table 2 and Table 3, respectively. At admission, he had a Laboratory Risk Indicator for Necrotizing Fasciitis (LRINEC) score of 4. Day 1 is described as the day of admission.

**Table 1:** Patient Vitals

Days (Day of admission=Day 1)	Vitals				
	Blood Pressure (Normal>90 mmHg/>60 mmHg)	Heart Rate (Normal<100 beats per minute [bpm])	Respirations (Normal <20 breaths per minute [bpm])	Temperature (Normal >97°F and <99°F or 36.1°C to 37.2°C)	SpO_2_% (Normal 95-100)
Day 1	112/51 mmHg	137 bpm	29 bpm	99.2°F (37.3°C)	93
Day 2	61/45 mmHg	133 bpm	25 bpm	98.1°F (36.7°C)	97

**Table 2:** Patient Lab Values

Labs	Patient Values		Low Normal	High Normal
Anion Gap	16 mEq/L	14 mEq/L	4 mEq/L	20 mEq/L
Glucose	102 mg/dL	225 mg/dL	70 mg/dL	104 mg/dL
BUN	34 mg/dL	44 mg/dL	8 mg/dL	21 mg/dL
Sodium	142 mEq/L	129 mEq/L	136 mEq/L	145 mEq/L
Creatinine	2.6 mg/dL	3.8 mg/dL	0.9 mg/dL	1.5 mg/dL
Glomerular Filtration Rate (GFR)	26.1 mL/min/1.73 m²	16.8 mL/min/1.73 m²	75 mL/min/1.73 m²	116 mL/min/1.73 m²
Calcium	8.4 mg/dL	6.6 mg/dL	8.8 mg/dL	10.2 mg/dL
Alkaline phosphatase	61 U/L	68 U/L	45 U/L	122 U/L
Albumin	2.8 g/dL	2.3 g/dL	3.5 g/dL	5 g/dL
Total protein	5.7 g/dL	4.9 g/dL	6.3 g/dL	8.3 g/dL
C-reactive protein (CRP)	6.17 mg/L	25.9 mg/L	0 mg/L	0.5 mg/L
Lactic Acid	6.4 mmol/L	6.0 mmol/L	0 mmol/L	2.2 mmol/L
Days (Day of admission=Day 1)	Day 1	Day 2		

**Table 3:** Patient Complete Blood Count

Complete Blood Count	Patient Values		Low Normal	High Normal
White Blood Cell Count	26.7 cells/µL	40.7 cells/µL	4.5 cells/µL	10.9 cells/µL
Red Blood Cell Count	4.91 cells/µL	4.84 cells/µL	4.7 cells/µL	6.1 cells/µL
Hemoglobin	15.2 g/dL	14.9 g/dL	14 g/dL	18 g/dL
Hematocrit	47.3%	46.4%	42%	52%
Mean Corpuscular Value	96 μm³	96 μm³	80 μm³	98 μm³
Neutrophils	25.3 k/cumm	38.0 k/cumm	1.4 k/cumm	10 k/cumm
Lymphocytes	0.2 k/cumm	0.4 k/cumm	1.2 k/cumm	3.4 k/cumm
Monocytes	0.6 k/cumm	0.3 k/cumm	0 k/cumm	1 k/cumm
Eosinophils	0.01 k/cumm	0.23 k/cumm	0 k/cumm	0.45 k/cumm
Basophils	0.2 k/cumm	0 k/cumm	0 k/cumm	1 k/cumm
Immunoglobulin	0.47 k/cumm	1.66 k/cumm	0 k/cumm	0.01 k/cumm
Days (Day of admission=Day 1)	Day 1	Day 2		

**Figure 2:**
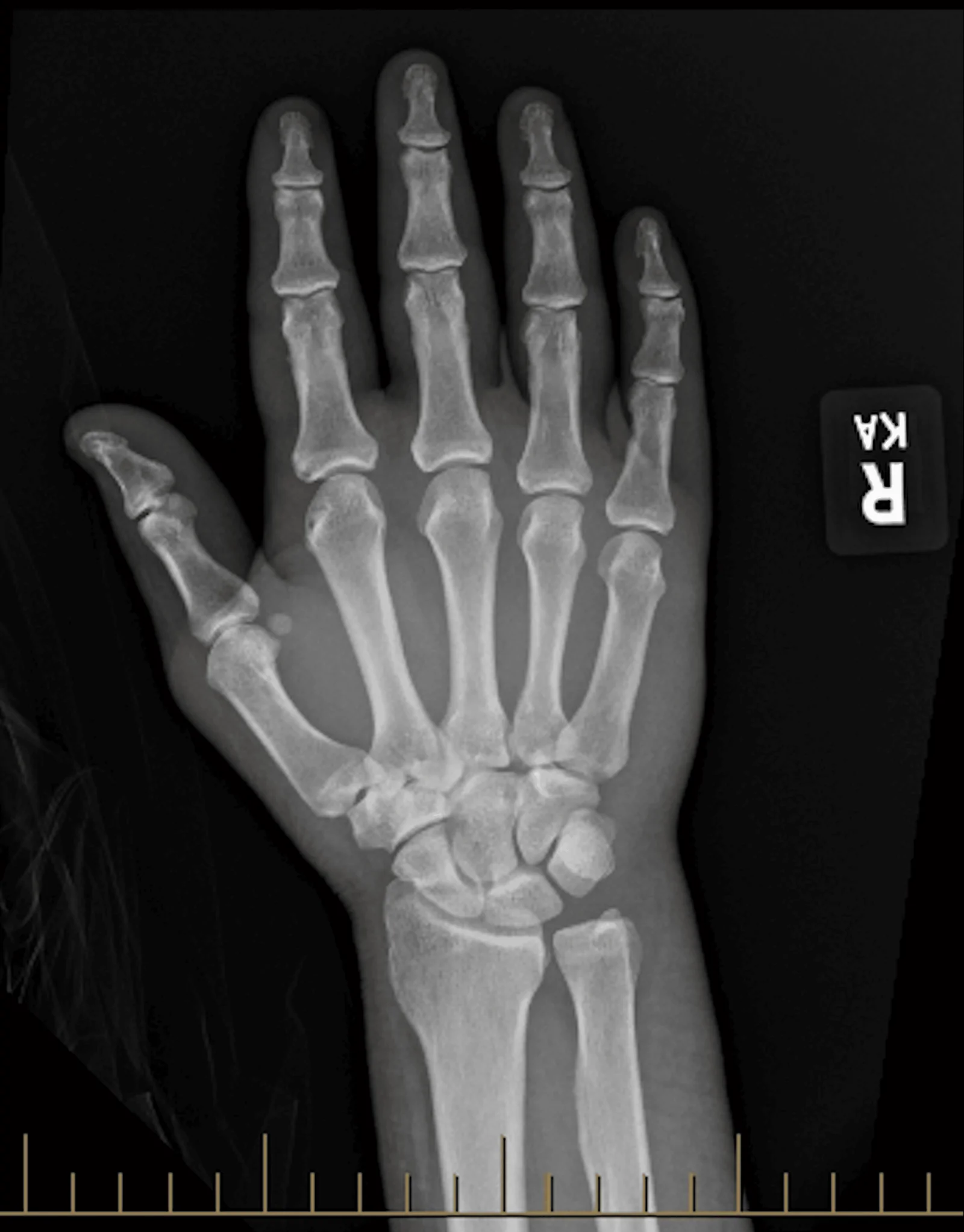
X-ray of the right hand showing no bony involvement on Day 1

**Figure 3:**
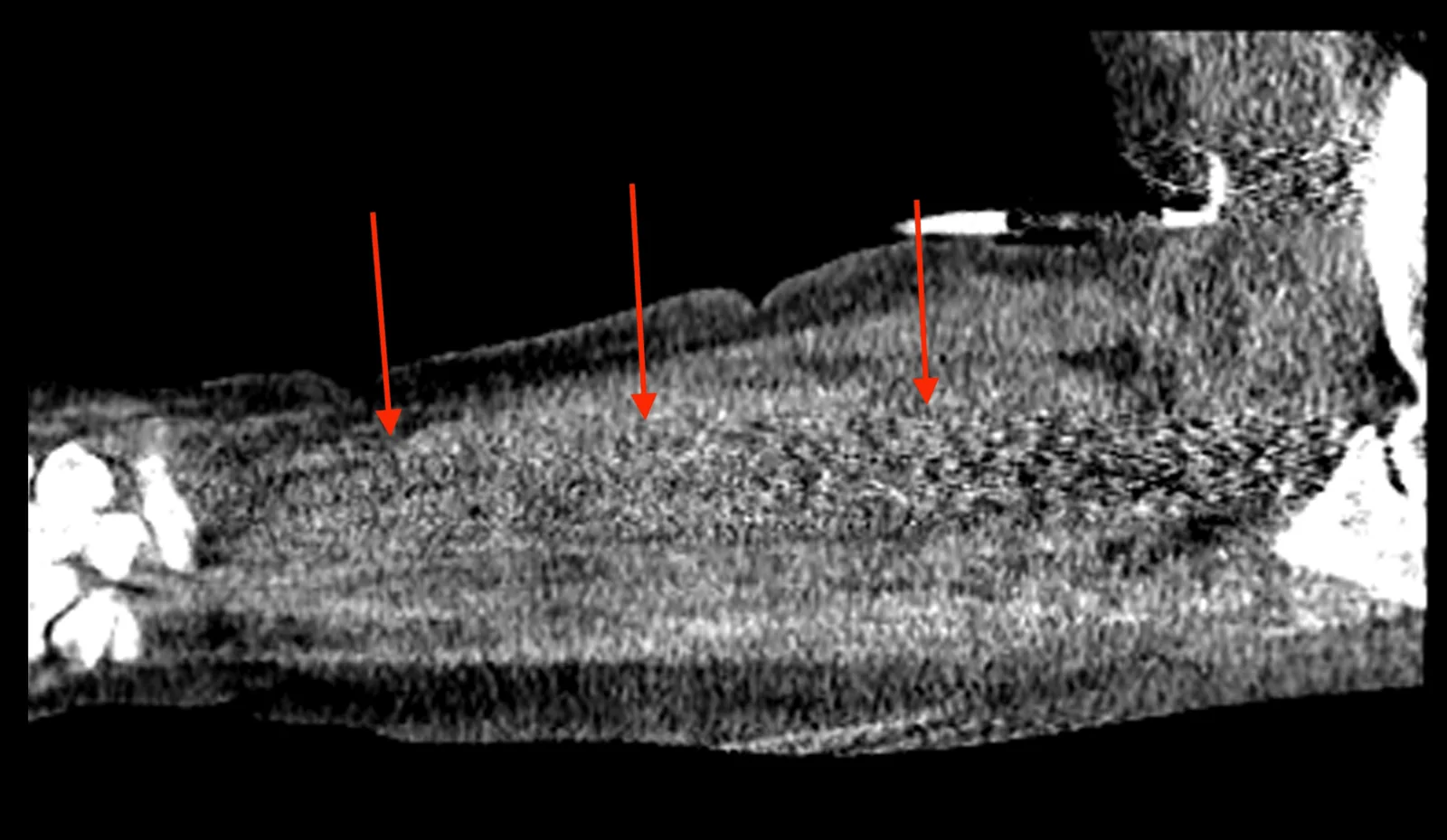
Coronal CT scan of the right forearm with evidence of severe soft tissue infection on Day 1. The red arrows highlight soft tissue swelling within the wound, supporting a diagnosis of necrotizing fasciitis. CT=computed tomography

**Figure 4:**
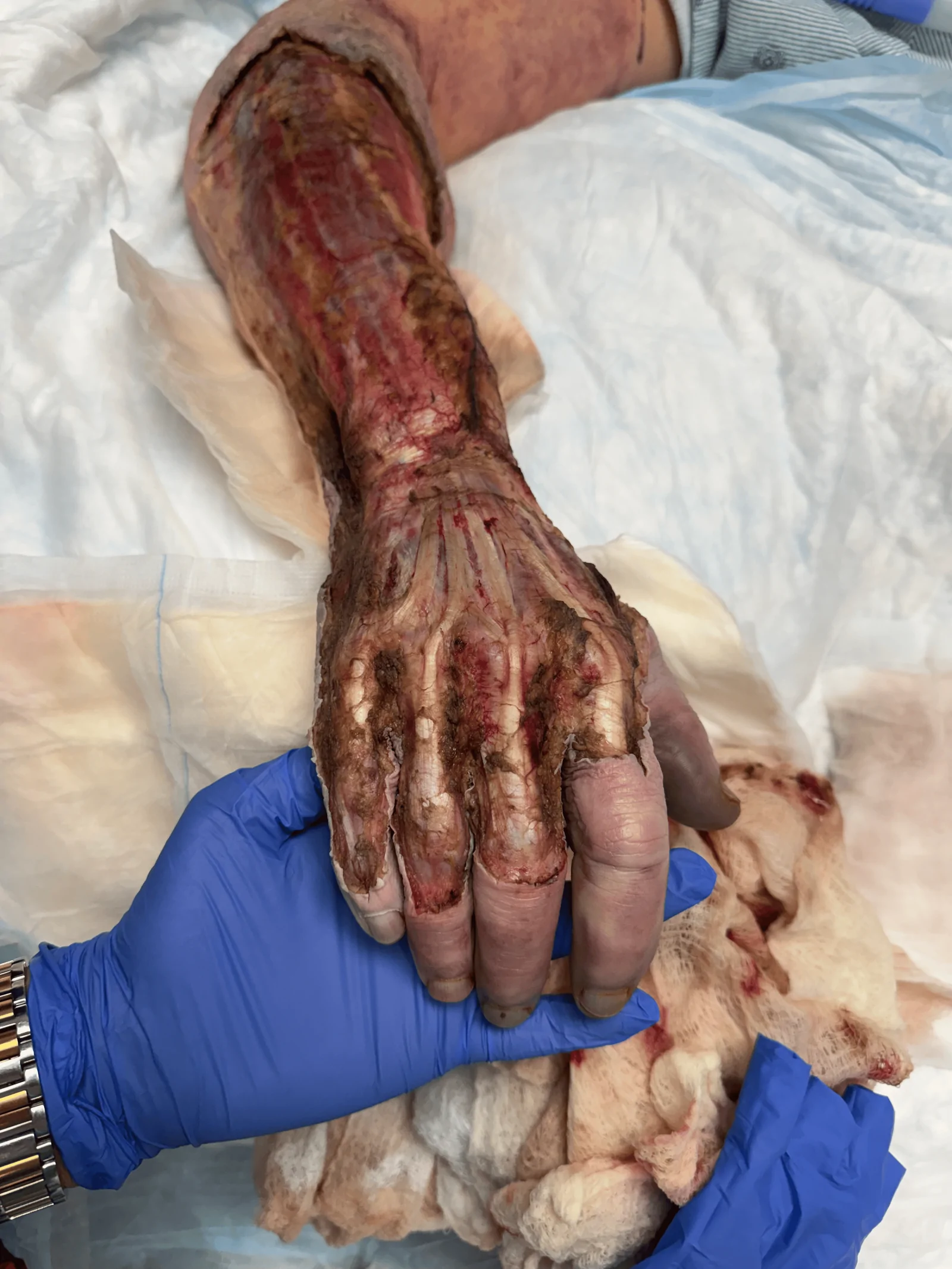
Right upper extremity at 8 am on Day 2 after the first debridement.

**Figure 5:**
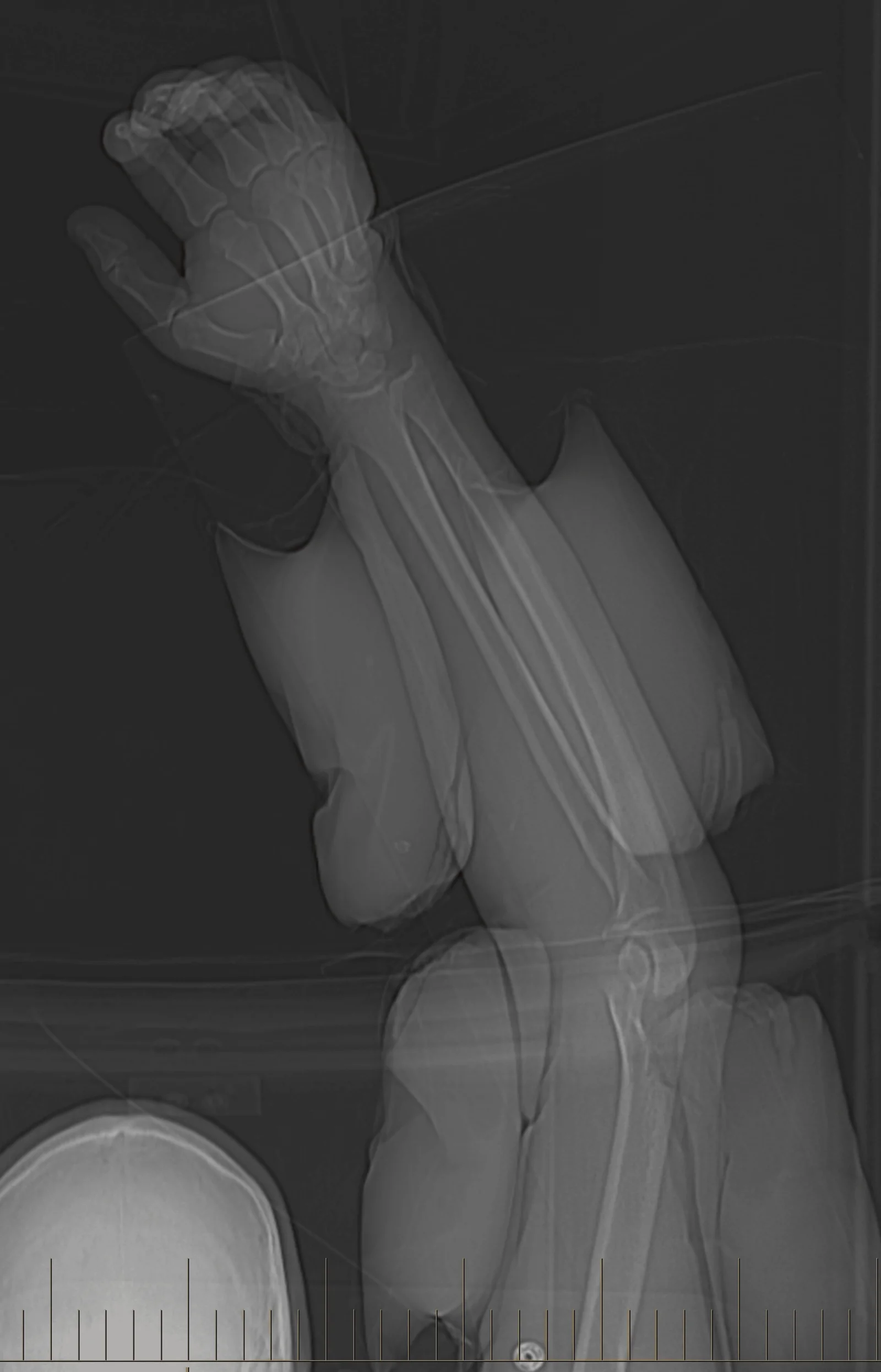
AP view X-ray of the right upper extremity showing no bony involvement

**Figure 6:**
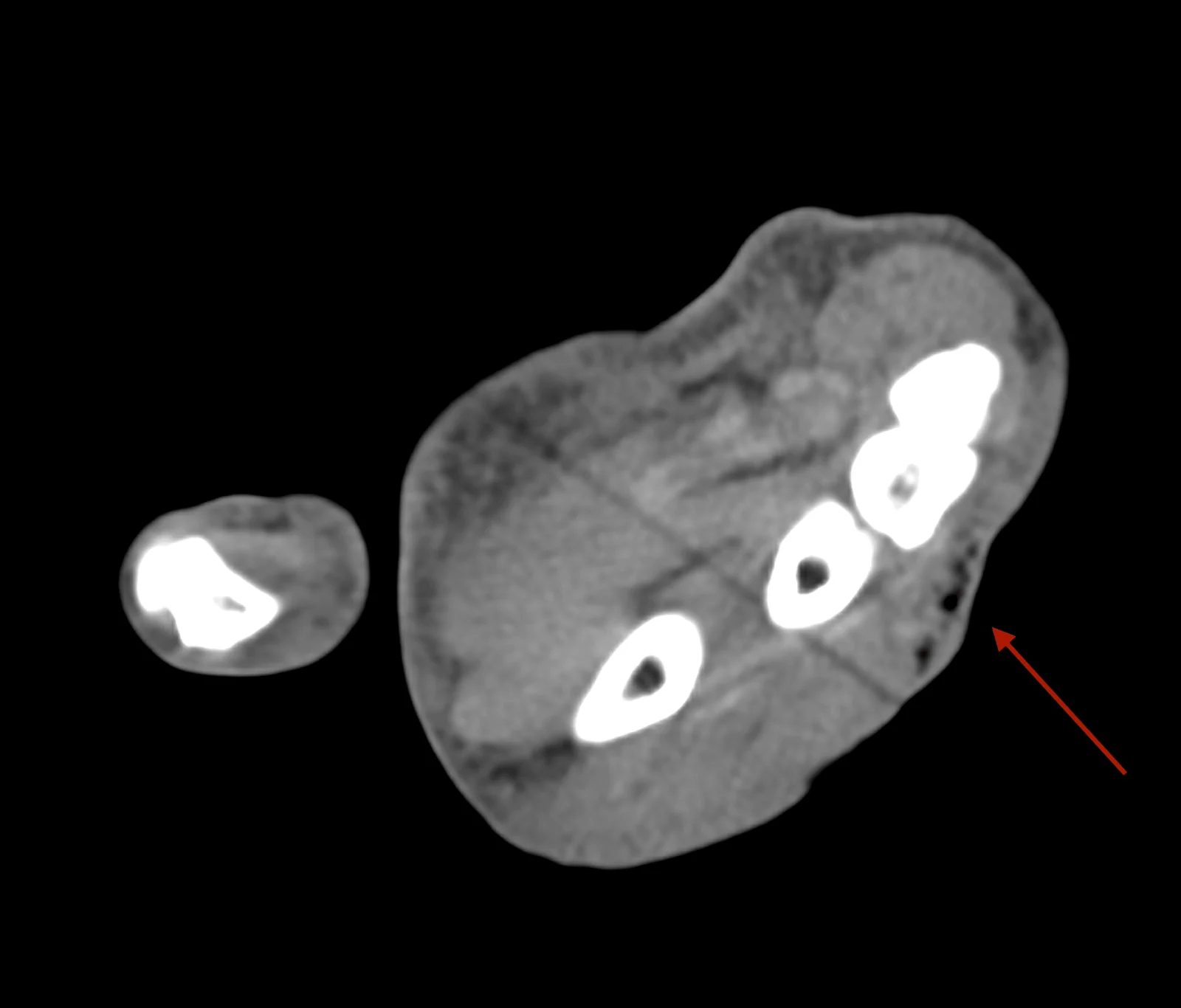
Transverse CT scan without contrast of the right hand. The red arrow points to emphysema over the dorsal aspect of the hand. CT=computed tomography

He was admitted to the hospital, sedated, intubated, and put on a number of vasopressors. Imaging was obtained at admission (Day 1), which showed no bony involvement (Figures 2-3). Urgent surgery consultation resulted in surgical debridement with loss of vascular status on that day. The following morning on Day 2, the patient continued to deteriorate and show signs of sepsis and multiorgan failure (Figure 4). At 24 hours after admission (Day 2), his LRINEC score was 11. He underwent a second surgical debridement. On Day 3, further imaging was obtained, which showed no bony involvement, soft tissue edema, and emphysema (Figure 5, Figure 6, and Video 1).

**Video 1:** Transverse CT scan without contrast of the right upper extremity on Day 3.

Due to the poor vascular outcome of the debridement and continued infection, the decision was made by a multidisciplinary team of an orthopedic surgeon, general surgeon, and internal medicine physician to perform a right upper extremity amputation above the elbow at the distal third of the humerus shaft on Day 4. A wound vacuum-assisted closure (VAC) was attached to the amputation stump. The patient had minimal blood loss and was sent to the post-anesthesia care unit (PACU) in stable condition. Wound and blood cultures returned positive for *Streptococcus pyogenes*.

He remained intubated and sedated for two weeks. During that time, he developed *Streptococcus pyogenes* pneumonia, which he was treated for in the ICU. Three weeks after his initial presentation to the ER, he was discharged to an outpatient rehabilitation facility.

The day after he was discharged, the patient experienced shortness of breath, chest pain, and generalized malaise. He became hypertensive, and he was admitted to the ICU at a tertiary care center. Orthopedic surgery was consulted for possible recurrent necrotizing fasciitis. The consult found no evidence of recurrent necrotizing fasciitis and recommended continued intravenous cefepime, clindamycin, and vancomycin.

**Figure 7:**
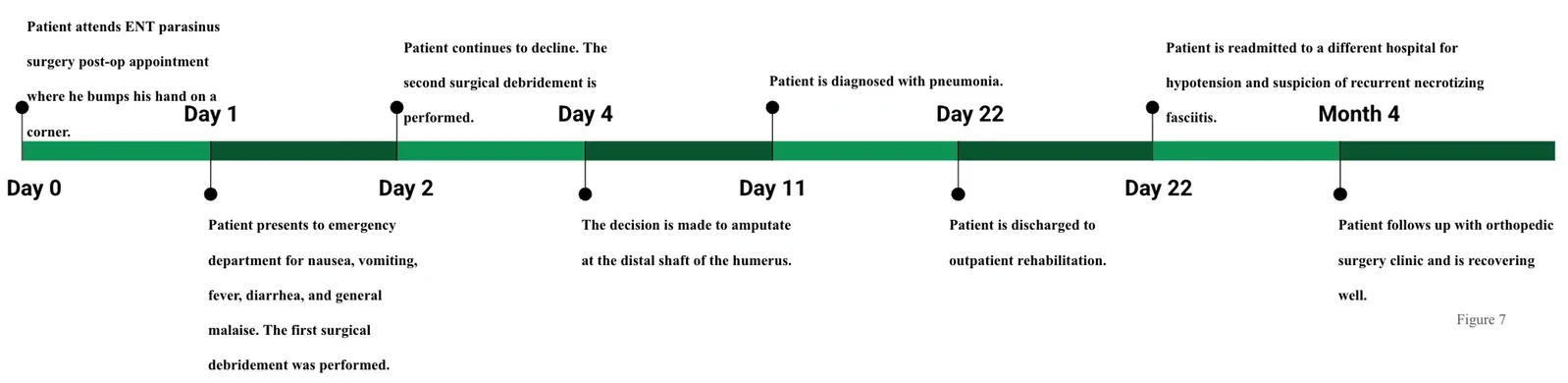
Timeline of clinical course Image created on Google Slides.

At 4-month follow-up, the patient reported that he was overall doing well and continuing wound care. His clinical timeline is shown in Figure 7.

## Discussion

This patient’s case had a difficult management course and required a multidisciplinary team to establish the best course of action. Amputation is considered a last-line therapy for NF and is used sparingly due to its life-altering effects. The Journal of the American Medical Association Surgery recommends that amputation be considered when (1) source control cannot be achieved with debridement, (2) reconstruction is unfeasible, or (3) functional status is expected to be better with amputation than reconstruction [8]. This case demonstrated a rapidly deteriorating course in which amputation became the best possible option for patient mortality within 48 hours of presentation and 72 hours of injury. This patient’s case was especially challenging due to his immunocompromised status.

In Keung et al.’s study of necrotizing soft tissue infections in immunocompetent versus immunocompromised individuals, immunocompromised individuals had a mortality rate of 76.1% compared to 40.6% in immunocompetent individuals [9]. Because of this stark increase in mortality, clinicians should maintain a high index of suspicion for necrotizing fasciitis in immunocompromised individuals. If a physician is unsure of the diagnosis, serial examinations should be performed every 4 to 6 hours. Extension of erythema beyond the initial border, necrotic or violaceous skin changes, or clinical worsening are strongly suggestive of necrotizing fasciitis and indicate surgical exploration.

Since first introduced as a potential diagnostic tool by Wong et al., LRINEC has been widely accepted with a cutoff value of ≥6 [10]. A meta-analysis done by Tarricone et al. showed that LRINEC’s strongest diagnostic performance was in negative predictive value and specificity of 83.17% and 89.9%, respectively [11]. The prognostic reliability of the LRINEC score found that a score >6 was statistically significant and predicted a higher number of necessary operations, but it is overall not a widely accepted prognostic tool [12].

A history of cancer, being transferred from an outside hospital, intravenous drug abuse, involvement of the anterior abdominal wall, lower admission systolic (median=107.5) and diastolic (median=52) blood pressure, and lower admission temperature were predictors of mortality in necrotizing fasciitis patients in a study done by Yaghoubian et al. [13]. This patient had a history of cancer and a lower systolic and diastolic blood pressure on one day after admission (Day 2). Additionally, Yaghoubian et al. found that when patients had a combination of lactic acid <54.1 and a sodium <135, the mortality was 19% [13]. This patient had a lactic acid of 6.0 and a sodium of 129 on Day 2. These predictors support a severe case of necrotizing fasciitis that required aggressive treatment.

The interaction of an autoimmune disease and stem cell transplant on the long-term immune status of an individual has conflicting evidence. Tanaka et al.’s analysis showed no statistically significant increase in nonrelapse mortality [14]. Other studies found that a pre-existing autoimmune disease had a modest impact on nonrelapse mortality and survival [15, 16].

## Conclusions

The decision to amputate is a serious, life-changing medical decision that requires input from a multidisciplinary team of physicians. This case demonstrates a case of rapidly progressing, fulminant necrotizing fasciitis and possible sequelae of immunocompromised status from both his steroid shot and remote history of stem cell transplant. This case supports that early recognition, aggressive multidisciplinary management, and a low threshold for definitive source control are vital to decreasing mortality.
